# Effects of powdered Montmorency tart cherry supplementation on an acute bout of intense lower body strength exercise in resistance trained males

**DOI:** 10.1186/s12970-015-0102-y

**Published:** 2015-11-16

**Authors:** Kyle Levers, Ryan Dalton, Elfego Galvan, Chelsea Goodenough, Abigail O’Connor, Sunday Simbo, Nicholas Barringer, Susanne U. Mertens-Talcott, Christopher Rasmussen, Mike Greenwood, Steven Riechman, Stephen Crouse, Richard B. Kreider

**Affiliations:** Department of Health and Kinesiology, Exercise and Sport Nutrition Laboratory, Texas A&M University, College Station, TX 77843-4243 USA; United States Military-Baylor University Graduate Program in Nutrition, AMEDD Center and School, Fort Sam Houston, United States Military, San Antonio, TX 78234 USA; Department of Nutrition and Food Science, Institute for Obesity Research and Program Evaluation, Texas A&M University, College Station, TX 77843-4243 USA; Department of Health and Kinesiology, Human Countermeasures Laboratory, Texas A&M University, College Station, TX 77843-4243 USA; Department of Health and Kinesiology, Applied Exercise Science Laboratory, Texas A&M University, College Station, TX 77843-4243 USA

**Keywords:** Tart cherry, Resistance exercise, Recovery, Antioxidants, Anti-inflammatory, Muscle damage

## Abstract

**Background:**

The purpose of this study was to examine whether short-term ingestion of a powdered tart cherry supplement prior to and following intense resistance-exercise attenuates muscle soreness and recovery strength loss, while reducing markers of muscle damage, inflammation, and oxidative stress.

**Methods:**

Twenty-three healthy, resistance-trained men (20.9 ± 2.6 yr, 14.2 ± 5.4 % body fat, 63.9 ± 8.6 kg FFM) were matched based on relative maximal back squat strength, age, body weight, and fat free mass. Subjects were randomly assigned to ingest, in a double blind manner, capsules containing a placebo (P, *n* = 12) or powdered tart cherries [CherryPURE^®^] (TC, *n* = 11). Participants supplemented one time daily (480 mg/d) for 10-d including day of exercise up to 48-h post-exercise. Subjects performed ten sets of ten repetitions at 70 % of a 1-RM back squat exercise. Fasting blood samples, isokinetic MVCs, and quadriceps muscle soreness ratings were taken pre-lift, 60-min, 24-h, and 48-h post-lift and analyzed by MANOVA with repeated measures.

**Results:**

Muscle soreness perception in the vastus medialis (¼) (*p* = 0.10) and the vastus lateralis (¼) (*p* = 0.024) was lower in TC over time compared to P. Compared to pre-lift, TC vastus medialis (¼) soreness was significantly attenuated up to 48-h post-lift with vastus lateralis (¼) soreness significantly lower at 24-h post-lift compared to P. TC changes in serum creatinine (*p* = 0.03, delta *p* = 0.024) and total protein (*p* = 0.018, delta *p* = 0.006) were lower over time and smaller from pre-lift levels over time compared to P Significant TC group reductions from pre-lift levels were found for AST and creatinine 48-h post-lift, bilirubin and ALT 60-min and 48-h post-lift. No significant supplementation effects were observed for serum inflammatory or anti-inflammatory markers. None of the free radical production, lipid peroxidation, or antioxidant capacity markers (NT, TBARS, TAS, SOD) demonstrated significant changes with supplementation. Changes in TC whole blood lymphocyte counts (*p* = 0.013) from pre-lift were greater compared to P, but TC lymphocyte counts returned to pre-lift values quicker than P.

**Conclusion:**

Short-term supplementation of Montmorency powdered tart cherries surrounding a single bout of resistance exercise, appears to be an effective dietary supplement to attenuate muscle soreness, strength decrement during recovery, and markers of muscle catabolism in resistance trained individuals.

## Background

A large volume, high intensity strength training workout activates a load-induced stress response characterized by structural muscle damage, oxidative stress, and inflammation that facilitates the release of intramuscular proteins into systemic circulation that are usually associated with cardiovascular dysfunction, invasive surgery, and disease [1–4]. The repetitive nature of muscle contractions during bouts of high intensity exercise will induce muscular injury as a result of ultrastructural disruptions [3, 5–7] that ultimately lead to a muscular repair sequence of events: degeneration, inflammation, regeneration, and fibrosis [3, 8]. Muscle soreness following exercise is not the direct result of inflammation, but rather a product of high nociceptor and mechanoreceptor sensitivity to the potent chemicals and by-products released during muscular degeneration [5, 9, 10]. Millions of people, including athletes, use non-steroidal anti-inflammatory drugs (NSAIDs) to help reduce pain and inflammation [3, 5]. NSAID use mitigates the inflammatory response via non-specific inhibition of the cyclooxygenase (COX-1 and COX-2) enzymes that regulate production of inflammatory-stimulating prostaglandins [3, 5, 11]. However, NSAIDs remain controversial as some research has demonstrated that muscle protein synthesis and the function of satellite cells in skeletal muscle hypertrophy are compromised when COX enzymes are inhibited [12–16], while other studies have found no NSAID effect on post-exercise anabolic processes within muscle [17–19].

Due to the debate surrounding the use of NSAIDs in sport and exercise applications, performance nutrition research has more recently shifted focus toward phytochemical-containing fruits and other functional foods that seem to provide a beneficial anti-inflammatory and antioxidant effect [20, 21]. A wide variety of antioxidant and polyphenol-containing functional foods such as purple sweet potatoes [22–24], beet root juice [25–27], cranberries [28, 29], and blueberries [30, 31] have verified health, performance-enhancing, and exercise recovery benefits. However, compared to other functional foods, the high anthocyanin content of both tart (e.g. Montmorency cherries) and sweet (e.g. Bing cherries) [21, 32–34] have proven beneficial in health [35–38], inflammatory-related disease states (e.g. cardiovascular disease, diabetes, osteoarthritis, gout) [35, 36, 38–40], and sleep quality [41, 42]. Clinical supplementation success with cherries, particularly tart cherry whole fruit, concentrates and cultivar juice blends, spurred an increase in exercise-based research to prove beneficial effects in mitigating muscle damage, oxidative stress, inflammation, and muscle pain with an impetus to increase performance [21, 43, 44].

We are aware of only two studies that have evaluated the effects of tart cherry supplementation on responses to resistance-based exercise. The first study examined 8-d of tart cherry cultivar-blended juice supplementation on exercise-induced muscle pain surrounding a bout of 40 maximal eccentric elbow flexion contractions (2 sets × 20 repetitions) [20]. As a result of tart cherry supplementation, strength losses and exercise-induced muscle pain were attenuated compared to a placebo over 4-d of maximal eccentric elbow flexion exercise [20]. No hematological analysis was performed to decipher potential physiological effects of supplementation. A second study examined the effects of 10-d tart cherry concentrate supplementation on physiological markers of muscle damage, oxidative stress, inflammation, muscle function, and muscle pain surrounding an intensive single-leg 100 repetition knee extensor training session (10 sets × 10 repetitions) [4]. Similar to the first study, functional recovery of isometric muscle strength was greater as a result of tart cherry supplementation compared to placebo [4]. Hematological analysis demonstrated reduced oxidative stress following the leg extension protocol that supported the beneficial functional recovery results in the tart cherry trial [4]. Interestingly, a recent endurance-based study with blended Montmorency tart cherry juice supplementation evaluating stress, inflammation, and upper respiratory symptoms following a marathon run demonstrated an attenuation of post-race incidence and severity of upper respiratory tract symptoms and inflammation (serum CRP) [45]. The primary aim of the current study was to determine if this powdered supplement derived from tart cherry skins would promote similar attenuation of muscle soreness, strength losses, and oxidative damage as the tart cherry cultivar-blended juice and juice concentrate supplemented in the two previous resistance exercise studies. The secondary objective of this study was to determine whether short-term (10-d) ingestion of the powdered tart cherry formulation prior to and following a lower body strength exercise protocol of high volume and intensity will effectively reduce markers of muscle damage, muscle soreness, inflammation, oxidative stress, and attenuate strength losses during subsequent exercise performance.

## Methods

### Subjects

**Fig. 1:**
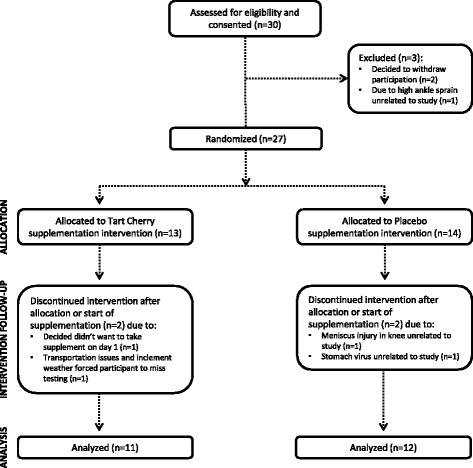
Consort diagram breakdown of the subject population as it pertains to the various stages of study progression

Twenty-three healthy, resistance-trained males (20.9 ± 2.6 yr, 81.7 ± 10.3 kg 14.2 ± 5.4 % body fat, 63.9 ± 8.6 kg FFM) participated as subjects in this study. Subjects were recruited through paper and electronically distributed study advertisements at Texas A&M University. Entrance criteria required subjects to have been involved in a progressive resistance training program that included regular squat exercise for at least 6 months prior to study recruitment and be able to perform a standard barbell back squat in a Smith machine rack of at least 1.5 times their body weight. Proper performance of barbell back squat technique was determined by a National Strength and Conditioning Association (NSCA) Certified Strength and Conditioning Coach (CSCS) during baseline testing. Figure 1 demonstrates the breakdown of the subject population as it pertains to the study progression. Discontinuation of any subject participation was not related to any aspect of the supplementation or testing protocol.

All subjects signed informed consent documents and the study was approved by the Texas A&M University Institutional Review Board prior to any data collection. Subjects were not allowed to participate in this study if they reported any of the following: 1) any metabolic disorders or taking any thyroid, hyperlipidemic, hypoglycemic, anti-hypertensive, anti-inflammatory (e.g. NSAIDs), and/or androgenic medications; 2) history of hypertension, hepatorenal, musculoskeletal, autoimmune, and/or neurological disease(s); 3) allergy to cherries or any cherry components (e.g. polyphenols, anthocyanins, anthocyanidins).

### Experimental design

**Fig. 2:**
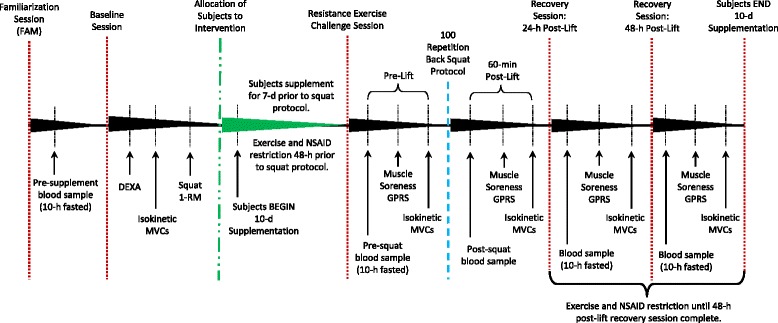
Experimental study design. DEXA = dual-energy X-Ray absorptiometer, MVC = maximal voluntary contraction, 1-RM = 1-repetition maximum, NSAID = non-steroidal anti-inflammatory drugs, GPRS = graphic pain rating scale, 7-d = 7-day, 48-h = 48-hour

Figure 2 shows the experimental design used in this study. The study was conducted in a randomized, double-blind, and placebo-controlled manner. All subjects eligible to participate in the study completed a morning familiarization (FAM) session where they were provided detailed information regarding the study design, testing procedures, and supplementation protocols. Informed consent, medical history, and endurance training history questionnaires were also completed during the FAM session. A research nurse reviewed medical history documents and performed a physical exam on each subject to ensure safety and participation eligibility. A fasting blood sample was taken at the end of the FAM session if the subject met entrance criteria. Subjects were asked to not change their dietary habits in any way throughout the study (upon start of supplementation). This was monitored by subject documentation of dietary intake for 4-d (3 weekdays and 1 weekend day) of the first seven supplementation days prior to the resistance exercise challenge. Approximately 10-d prior to the resistance exercise intervention, eligible subjects returned to the lab for a morning baseline testing session to determine body weight, height, and body composition. The subjects then were familiarized with the maximal voluntary contraction (MVC) protocol using their dominant leg on an isokinetic knee extension dynamometer followed by a 5-min recovery before determination of their barbell back squat 1-repetition maximum (1-RM) in a Smith machine rack. Following baseline measurements, subjects were matched based on relative maximal back squat strength, fat free mass, body weight, and age accompanied by a random separation into two groups: 1) a placebo group or 2) a powdered tart cherry group.

Subjects were instructed to begin supplementation 7-d prior to the resistance exercise challenge (Day 0). Subjects were asked to fast overnight for 10-h to account for diurnal variation as well as refrain from exercise and consumption of NSAIDs for 48-h prior to all testing days. Due to the large variety of NSAIDs commonly available with half-life data ranging from approximately 1.6 to 30-h [46], the 48-h restriction was implemented to help reduce any potential concurrent effects. Exercise logs were not implemented to track subject activity patterns over the study protocol. On the day of the resistance exercise challenge, subjects reported to the lab where body weight, resting heart rate, and resting blood pressure were measured. Subjects then donated a fasting venous blood sample (approximately 20 ml) using standard clinical procedures and rated perceptions of muscle soreness to a standardized application of pressure on their dominant thigh at three designed locations using a graphic pain rating scale (GPRS). The subjects then completed the MVC protocol using their dominant leg on an isokinetic knee extension dynamometer followed by a 5-min recovery before the barbell back squat protocol utilizing a Smith machine rack was completed with a Tendo power and speed analyzer attached to the bar. Fasting (except 60-min post-lift) blood samples, GPRS ratings of quadriceps muscle soreness, and the isokinetic leg extension MVC protocol were completed at 60-min, 24-h and 48-h of post-lift recovery. The last day of supplementation correlated with 48-h post-lift recovery.

### Exercise protocol

#### Isokinetic knee extension maximal voluntary contractions

At baseline, before the squat exercise, 60-min after the squat exercise, 24-h and 48-h post-lift exercise, subjects performed an isokinetic knee extension MVC test using their dominant leg on a Kin-Kom 125AP Isokinetic Dynamometer (*Chattanooga-DJO Global Inc., Vista, CA, USA*). During the baseline familiarization of the isokinetic leg extension MVC protocol, standard procedures were used to determine and record proper subject positioning on the Kin-Kom to ensure accurate repeatability of the testing protocol across all five sessions. The subject warmed-up on the isokinetic dynamometer by performing three-sets of five repetitions of dominant knee extension/flexion at 50 % of their MVC (determined during the FAM) with 1-min recovery between sets. After the 1-min recovery following the third warm-up set, the subject performed 3 MVCs in succession with no rest between contractions. Subjects were allotted a 5-min recovery between the isokinetic MVC and barbell back squat protocols. Test to test variability of performing this test yielded average C_V_ values of ±8.47 % with a test retest correlation of *r* = 0.88.

#### Barbell back squat 1-repetition maximum determination

At baseline, following the 5-min recovery after the isokinetic MVCs, subjects performed three-sets of five repetitions of a barbell back squat on a Smith machine rack (*Life Fitness, Schiller Park, IL, USA, model SSM)* at 50 % of their anticipated 1-RM with 2-min recovery between sets. Following the warm-up protocol, the barbell back squat 1-RM was determined on the Smith machine rack using a standard 1-RM protocol defined by the NSCA with 2-min recovery periods between 1- repetition sets of increasing weight. All 1-RM determinations were made within 3–5 sets beyond the warm-up. In order to successfully complete each set, the subject was required to reach thigh parallel depth as determined by a NSCA Certified Strength and Conditioning Coach (CSCS) providing a verbal “up” cue during spotting.

#### Barbell back squat exercise protocol

On the day of the resistance exercise challenge, following the 5-min recovery after the isokinetic MVCs, subjects performed one warm-up set of ten repetitions of the barbell back squat at 50 % 1-RM (determined during baseline). After 3-min recovery, the subjects performed ten sets of ten repetitions of a barbell back squat at 70 % 1-RM with 3-min recovery between sets. If necessary, the barbell back squat resistance was adjusted such that each subject completed the 100 repetition (10 sets × 10 repetitions) volume to thigh parallel depth, keeping the training volume consistent across the study population. This protocol is closely replicated after the repeated leg extension exercise implemented previously by Bowtell et al. [4]. A Tendo Power and Speed Analyzer (*TENDO Sports Machines, Trencin, Slovak Republic, model PSA310*) was attached to the bar during all 100 repetitions completed during the squat protocol to determine peak power, average force, and total work for each repetition. Following the squat protocol, subjects were only permitted to stretch with limited ambulation until the 60-min post-lift testing protocol was completed.

### Supplementation protocol

Subjects were assigned in a double-blinded and randomized manner to ingest either a rice flour placebo (P, *n =* 12) or powdered tart cherry (TC, *n =* 11). Subjects were matched into one of the two groups according to relative maximal back squat strength, fat free mass, body weight, and age. Subjects were instructed to ingest 480 mg of the supplements with breakfast at 0800 7-d prior to, the day of, and 2-d following the resistance exercise challenge for a total supplementation timeline of 10-d. The tart cherry supplements contained 480 mg of freeze dried Montmorency tart cherry skin powder derived from tart cherry skins obtained after juicing (*CherryPURE™ Freeze Dried Tart Cherry Powder, Shoreline Fruit, LLC, Transverse City, MI, USA*). Prior analytical testing conducted in 2012 by Atlas Bioscience (*Tuscon, AZ,* USA) demonstrated that 290 mg of *CherryPURE™* provides approximately 600 mg of phenolic compounds and 40 mg of anthocyanins, which is equivalent to consuming 10.5 fluid ounces of tart cherry juice. The supplements were prepared for distribution by Shoreline Fruit, LLC and sent to Advanced Laboratories (*Salt Lake City, UT, USA*) to quantify total phenolic compound and anthocyanin content of the powdered tart cherry supplements. Supplements were prepared in standardized color capsules and packaged in generic bottles by Shoreline Fruit, LLC for double blind administration.

### Procedures

#### Dietary inventories

Within the first 7-d of supplementation, subjects were instructed to record all food and fluid intake over a 4-d period (3 weekdays, 1 weekend day), which was reflective of their normal dietary intake. Dietary inventories were then reviewed by a registered dietician and analyzed for average energy, macronutrient, and dietary antioxidant intake using ESHA Food Processor (*Version 8.6*) Nutritional Analysis software (*ESHA Research Inc., Salem, OR, USA*).

#### Anthropometrics and body composition

At the beginning of every testing session, subjects had their height and body mass measured according to standard procedures using a Healthometer Professional 500KL (*Pelstar LLC, Alsip, IL, USA)* self-calibrating digital scale with an accuracy of ± 0.02 kg. Whole body bone density and body composition measures (excluding cranium) were determined with a Hologic Discovery W Dual-Energy X-ray Absorptiometer (DEXA; *Hologic Inc., Waltham, MA, USA*) equipped with APEX Software (*APEX Corporation Software, Pittsburg, PA, USA*) by using procedures previously described [47, 48]. Mean test-retest reliability studies performed on male athletes in our lab with this DEXA machine have revealed mean coefficients of variation for total bone mineral content and total fat free/soft tissue mass of 0.31–0.45 % with a mean intraclass correlation of 0.985 [49]. On the day of each test, the equipment was calibrated following the manufacturer’s guidelines.

#### Muscle soreness perception measurement

Pressure application to the three specified areas of the quadriceps muscle group on the subject’s dominant leg was standardized to 50 N of pressure using a handheld Commander Algometer (*JTECH Medical, Salt Lake City, UT, USA*). Force was applied to each site of muscle contact with a cylindrical metal probe through a 1 cm diameter head. The standard amount of pressure was applied to the vastus lateralis (VL) at both 25 and 50 % of the distance between the superior border of the patella and the greater trochanter of the femur at the hip and to the vastus medalis (VM) at 25 % of the distance between the aforementioned landmarks. These three specific locations were measured and marked with a permanent marker on each subject during the initial muscle soreness perception measurement before the resistance exercise challenge. The participants were asked to maintain these three marked locations between testing sessions to avoid error with secondary measurement. The subject was seated in a reclined supine position and given the GPRS sheet to evaluate the perception of muscle soreness at each of the three quadriceps locations. The order of pressure application was standardized across all sessions and participants: 25 % VM, 25 % VL, and 50 % VL. The 50 N of pressure was applied to a relaxed quadriceps at each of the three locations using the algometer for a period of 3-sec to give the subject enough time to record their soreness evaluation on the GPRS. Perceptions of muscle soreness were recorded by measuring the distance (centimeters) of the participant mark on the GPRS from 0 cm (no pain). Test-to-test reliability for this protocol revealed a mean intraclass correlation of 0.90.

#### Blood collection

Subjects were required to fast (except 60-min post-lift) for 10-h prior to donating approximately four teaspoons (20 mL) of venous blood from an antecubital vein using standard phlebotomy procedures at each of the testing sessions. Blood analyzed for markers of muscle damage, oxidative stress, inflammation, and clinical chemistry panels were collected in two 7.5 mL BD Vacutainer^®^ serum separation tubes (*Becton, Dickinson and Company, Franklin Lakes, NJ, USA)*, left at room temperature for 15-min, and then centrifuged at 3500 rpm for 10-min using a standard, refrigerated (4 °C) bench top Thermo Scientific Heraeus MegaFuge 40R Centrifuge (*Thermo Electron North America LLC, West Palm Beach, FL, USA).* Serum supernatant was removed and stored at −80 °C in polypropylene microcentrifuge tubes for later analysis. Blood was also collected in a single 3.5 mL BD Vacutainer^®^ lavender top tube containing K_2_ EDTA (*Becton, Dickinson and Company, Franklin Lakes, NJ, USA)*, left at room temperature for 15-min, and refrigerated for approximately 3–4 h before subsequent complete blood count analysis.

#### Clinical chemistry analysis

Whole blood samples were analyzed for complete blood count with platelet differentials (hemoglobin, hematocrit, red blood cell counts (RBC), white blood cell counts (WBC), lymphocytes, granulocytes (GRAN), and mid-range absolute count (MID)) using an Abbott Cell Dyn 1800 (*Abbott Laboratories, Abbott Park, IL, USA*) automated hematology analyzer. Internal quality control for the Abbott Cell Dyn 1800 was performed using three levels of control fluids purchased from the manufacturer to calibrate acceptable standard deviation (SD) and coefficients of variation (C_V_) values for all aforementioned whole blood cell counts. Serum samples were analyzed using a Cobas c111 (*Roche Diagnostics GmbH, Indianapolis, IN, USA*) automated clinical chemistry analyzer that was calibrated and optimized according to manufacturer guidelines. This analyzer has been known to be highly valid and reliable in previously published reports [50]. Each serum sample was assayed for a standard partial metabolic panel [(aspartate aminotransferase (AST), alanine aminotransferase (ALT), and total bilirubin)] and clinical markers of protein and fatty acid metabolism [(uric acid, creatinine, blood urea nitrogen (BUN), total protein, and creatine kinase (CK)]. The internal quality control for the Cobas c111 was performed using two levels of control fluids purchased from the manufacturer to calibrate acceptable SD and C_V_ values for all aforementioned assays. Samples were re-run if the observed values were outside control values and/or clinical norms according to standard procedures.

#### Markers of anabolic/catabolic hormone status

Serum samples were assayed using standard commercially available enzyme-linked immunosorbent assay kits (ELISAs) for cortisol and testosterone (*ALPCO Diagnostics, Salem, NH, USA*) hormone analysis. Serum concentrations were determined calorimetrically using a BioTek ELX-808 Ultramicroplate reader (*BioTek Instruments Inc., Winooski, VT, USA*) at an optical density of 450 nm against a known standard curve using manufacturer recommended procedures. Samples were run in duplicate according to standard procedures to ensure validity of measurement. Test to test variability of performing these assays yielded average C_V_ values for the aforementioned markers of: CORT (±6.85 %), and TEST (±4.47 %) with a test retest correlation for the same markers of: CORT (*r =* 0.92), TEST (*r =* 0.98).

#### Markers of oxidative stress

Serum samples were assayed using standard commercially available ELISA kits for Superoxide Dismutase (SOD Activity Assay kit), Total Antioxidant Status (TAS, Antioxidant Assay kit), Thiobarbituric Acid Reactive Substance (TBARS, Malondialdehyde-MDA, TCA method kit) (*Cayman Chemical Company, Ann Arbor, MI, USA*), and Nitrotyrosine (*ALPCO Diagnostics, Salem, NH, USA*). Serum concentrations for SOD and Nitrotyrosine were determined calorimetrically using the previously stated instrumentation at the same optical density (450 nm) against a known standard curve using standard procedures. Serum concentrations for TAS were also determined calorimetrically using the aforementioned instrumentation at an optical density of 405 nm against a known standard curve using standard procedures. Lastly, serum concentrations for TBARS were also determined fluorometrically using a SpectraMax Gemini multimode plate reader (*Molecular Devices LLC, Sunnyvale, CA, USA*) at an excitation wavelength of 530 nm and an emission wavelength of 550 nm against a known standard curve using standard procedures. Samples were run in duplicate according to standard procedures to ensure validity of measurement. Test to test variability of performing these assays yielded average C_V_ values for the aforementioned markers of: SOD (±8.35 %), TAS (±14.24 %), TBARS (±8.30 %), and NT (±10.03 %) with a test retest correlation for the same markers of: SOD (*r =* 0.83), TAS (*r =* 0.85), TBARS (*r =* 0.94), and NT (*r =* 0.99).

#### Cytokine/Chemokine markers of inflammation

Serum markers of inflammation [(interleukin-1β (IL-1β), IL-2, IL-4, IL-5, IL-6, IL-7, IL-8, IL-10, IL-12p70, IL-13, tumor necrosis factor-α (TNF-α), interferon-γ (IFN-γ), and granulocyte-macrophage colony-stimulating factor (GM-CSF)] were measured by using a commercially available Milliplex MAP 13-Plex Human High Sensitivity T-Cell Magnetic Bead Panel kit (*EMD Millipore Corporation, St. Charles, MO, USA*) that was optimized for human serum samples. A minimum of 100 positive beads for each cytokine/chemokine was acquired with a Luminex MagPix instrument (*Luminex Corporation, Austin, TX, USA*). This instrument has been proven to be highly valid and reliable in previously published reports across many disciplines [51–54]. Manufacturer supplied controls were used to monitor the coefficients of variation. Samples were run in duplicate according to standard procedures to ensure validity of measurement. Test to test variability of performing these assays yielded average C_V_ values for the aforementioned markers of: IL-1β (±4.37 %), IL-2 (±5.52 %), IL-4 (±4.59 %), IL-5 (±4.30 %), IL-6 (±4.26 %), IL-7 (±5.05 %), IL-8 (±4.55 %), IL-10 (±5.05 %), IL-12p70 (±4.92 %), IL-13 (±4.99 %),TNF-α (±6.05 %), IFN-γ (±4.42 %), and GM-CSF (±5.22 %) with a test retest correlation for the same markers of: IL-1β (*r =* 0.98), IL-2 (*r =* 0.99), IL-4 (*r =* 0.99), IL-5 (*r =* 0.99), IL-6 (*r =* 0.99), IL-7 (*r =* 0.99), IL-8 (*r =* 0.99), IL-10 (*r =* 1.00), IL-12p70 (*r =* 1.00), IL-13 (*r =* 0.99), TNF-α (*r =* 0.95), IFN-γ (*r =* 1.00), and GM-CSF (*r =* 1.00).

#### Statistical analysis

Individual group and time data are presented throughout the text and in all tables as means (± SD), while group effects are presented as means (± SEM). All related variables were grouped and analyzed using repeated measures MANOVA in IBM SPSS Statistics Software version 22.0 for Windows (*IBM Corporation, Armonk, NY, USA*) to assess values observed and changes from pre-lift levels in response to the supplement administered. Post-hoc LSD pairwise comparisons were used to analyze any significance among groups where needed with Cohen’s d calculations employed to determine effect magnitude. Data was considered statistically significant when the probability of error was less than 0.05 and considered to be trending when the probability of error was less than 0.10. Statistical trends (*p* < 0.05 to *p* < 0.10) were noted as is common practice in studies with relatively small sample size [55].

## Results

### Subject baseline characteristics

A total of 23 healthy, resistance-trained men completed the protocol for this study. Participants were 20.9 ± 2.6 years, 81.7 ± 10.3 kg, 14.2 ± 5.4 % body fat, and 63.9 ± 8.6 kg fat free mass. Participant demographic data are presented in Table 1. One-way ANOVA revealed no significant differences (*p* > 0.05) in baseline demographic markers. No significant differences in back squat 1-RM (*p* = 0.70) and relative squat strength ratio (*p* = 0.85) were reported across groups, hence the groups were generally well matched. One-way ANOVA analysis also demonstrated no differences in total work performed (*p* = 0.79) across groups, indicating that similar work was performed during the exercise intervention and differences can likely be attributed to the nutritional intervention.

**Table 1:** Demographics by Study Group

Variable	Group	Mean	Group (SEM)	p-value
N	P	12	n/a	n/a
	TC	11	n/a	
	Total	23	n/a	
Age	P	20.58±1.78	0.51	0.593
	TC	21.18±3.34	1.01	
	Total	20.87±2.60	0.54	
Height (cm)	P	177±5.28	1.52	0.651
	TC	178±10.71	3.23	
	Total	177±8.17	1.70	
Weight (kg)	P	82.62±7.39	2.13	0.659
	TC	80.64±13.16	3.97	
	Total	81.67±10.35	2.16	
Baseline HR (bpm)	P	61.17±10.40	3.00	0.195
	TC	66.09±6.67	2.01	
	Total	63.52±8.98	1.87	
BMD (g/cm^2^)	P	1.16±0.10	0.03	0.389
	TC	1.12±0.14	0.04	
	Total	1.14±0.12	0.02	
LM (kg)	P	61.41±4.87	1.41	0.970
	TC	61.55±11.16	3.36	
	Total	61.48±8.27	1.73	
FFM (kg)	P	63.83±5.06	1.46	0.981
	TC	63.92±11.64	3.51	
	Total	63.87±8.62	1.80	
FM (kg)	P	11.81±5.44	1.57	0.595
	TC	14.10±13.58	4.09	
	Total	12.90±10.00	2.08	
Body Fat (%)	P	15.31±6.27	1.81	0.338
	TC	13.09±4.31	1.30	
	Total	14.25±5.42	1.13	
Back Squat 1-RM (lbs)	P	318±62	18.03	0.696
	TC	306±82	24.72	
	Total	313±71	14.81	
Relative Squat Strength Ratio	P	1.75±0.27	0.08	0.855
	TC	1.73±0.34	0.10	
	Total	1.74±0.30	0.06	
Workout Total Work (kJ)	P	0.68±0.14	0.04	0.798
	TC	0.70±0.23	0.07	
	Total	0.69±0.18	0.04	

Mean data expressed as means ± SD

*HR* heart rate, *BMD* bone mineral density, *LM* lean mass, *FFM* free-fat mass, *FM* fat mass, *1-RM* 1-Repetition maximum

### Nutritional intake and compliance

Nutritional intake was monitored over a 4-d period within the first 7-d of supplementation. Relevant nutritional components analyzed are listed in Table 2. No statistically significant interactions were observed across groups with respect to dietary intake.

**Table 2:** Dietary Analysis by Study Group

Variable	Group	Mean	Group (SEM)	p-value
Average Daily Caloric Consumption (kcal)	P	2617±721	208	0.605
	TC	2455±753	227	
	Total	2540±724	151	
Dietary Protein (g)	P	147.86±43.13	12.45	0.871
	TC	153.06±100.39	30.27	
	Total	150.35±74.28	15.49	
Dietary Carbohydrates (g)	P	244.47±62.58	18.07	0.460
	TC	223.52±70.90	21.38	
	Total	234.45±66.01	13.76	
Dietary Fat (g)	P	84.54±23.55	6.80	0.427
	TC	95.23±38.55	11.62	
	Total	89.65±31.35	6.54	
Dietary Beta-Carotene (mcg)	P	2954±3436	992	0.651
	TC	2111±5260	1586	
	Total	2551±4320	901	
Dietary Vitamin C [Ascorbic Acid] (mg)	P	80.79±65.99	19.05	0.199
	TC	50.56±38.41	11.58	
	Total	66.33±55.55	11.58	
Dietary Vitamin E [Alpha-Tocopherol] (mg)	P	5.91±6.04	1.74	0.910
	TC	6.24±7.44	2.24	
	Total	6.07±6.59	1.37	

Mean data expressed as means ± SD

### Muscle soreness perception assessment

**Fig. 3:**
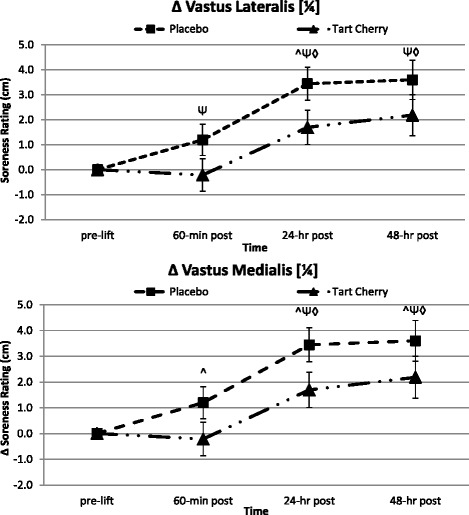
Perceptions of muscle soreness. Data expressed as means ± SE and significance indicated by the following super/subscripts: * indicates *p* < 0.05 p-level significance, § indicates *p* < 0.10 p-level significance. LSD post hoc analysis is indicated by the following superscripts: ^ represents *p* < 0.05 difference between groups, Ψ represents *p* < 0.05 difference from pre-lift, ◊ represents *p* < 0.05 difference from 60-min post, # represents *p* < 0.05 difference from 24-h post

Table 3 presents perceptions of muscle soreness across the resistance exercise protocol. The overall MANOVA analysis revealed a significant overall Wilks’ Lambda time (*p* < 0.001) interaction and a non-significant overall group x time effect (*p* = 0.20). Perception of muscle soreness in all three muscle testing locations irrespective of group significantly increased over time, peaking 48-h post-lift, indicating the onset of muscle soreness as a result of the lifting protocol. A significant difference between groups over time was found in vastus lateralis (¼) soreness perception (*p* = 0.024) where ratings increased by 55–170 % from pre-lift values in P, but only 35–104 % in TC over the recovery. Post-hoc analysis indicated a significantly attenuated increase in muscle soreness perception 24-h post-lift for TC supplementing subjects compared to P (see Fig. 3). Effects of supplementation tended to be different in the perception of vastus medialis (¼) soreness (*p* = 0.10) with significantly lower muscle soreness in TC versus P up to 48-h post-lift (see Fig. 3). Soreness perception ratings in the vastus medialis (¼) increased by 28–83 % from pre-lift values in P, but ranged from a 6 % decrease to only a 58 % increase in TC over the recovery.

**Table 3:** Quadriceps Muscle Soreness Perception

Variable	Group	Pre-Lift	60-min Post	24-hr Post	48-hr Post	Group Mean	p-value (GG)	p-value (WSC)
Algo I (cm)	P	4.34±2.52	5.53±3.15	7.78±2.93	7.94±2.92	6.40±0.76	*G=*0.139	
	TC	3.80±2.86	3.59±2.76	5.50±2.68	5.99±3.59	4.72±0.79	*T<*0.001*	*T*_*L*_ *<*0.001*
	Time Mean	4.07±0.56	4.56±0.62	6.64±0.59^Ψ◊^	6.96±0.68^Ψ◊^		*G X T=*0.236	*G X T*_*q*_ *=*0.172
Algo II (cm)	P	2.44±1.60	3.76±2.24^Ψ^	6.60±2.99^^Ψ◊^	6.20±3.64^Ψ◊^	4.75±0.69	*G=*0.773	
	TC	2.98±2.70	4.03±2.93^Ψ^	4.76±3.03^Ψ^	6.07±3.67^Ψ◊#^	4.46±0.72	*T<*0.001*	*T*_*L*_ *<*0.001*
	Time Mean	2.71±0.46	3.90±0.54^Ψ^	5.68±0.63^Ψ◊^	6.14±0.76^Ψ◊^		*G X T=*0.187	*G X T*_*q*_ *=*0.024*
Algo III (cm)	P	3.25±2.65	3.42±3.24	6.55±3.80	6.18±3.36	4.85±0.81	*G=*0.609	
	TC	2.68±2.67	3.76±3.18	4.89±3.87	5.64±3.74	4.24±0.85	*T<*0.001*	*T*_*L*_ *<*0.001*
	Time Mean	2.96±0.55	3.59±0.67	5.72±0.80^Ψ◊^	5.91±0.74^Ψ◊^		*G X T=*0.427	*G X T*_*L*_ *=*0.690

Individual group and time data expressed as means ± SD, while group effects are presented as means ± SEM. Data represents the participant soreness perception in the quadriceps muscle group at each test session during the 10 day intervention. MANOVA analysis revealed overall Wilks' Lambda time (p<0.001) and group x time (p=0.199). Univariate ANOVA p-levels from MANOVA analysis are presented for each variable. Univariate ANOVA p-levels are listed first by the Greenhouse-Geisser (GG) analysis and then by the within-subjects contrasts (WSC) to demonstrate the potential shape of the time or group x time interaction with significance indicated by the following super/subscripts: * indicates p<0.05 p-level significance, § indicates p<0.10 p-level significance. LSD post hoc analysis is indicated by the following superscripts: ^ represents p<0.05 difference between groups, Ψ represents p<0.05 difference from pre-lift, ◊ represents p<0.05 difference from 60-min post, # represents p<0.05 difference from 24-hr post. Algo I = Algometer location #1: Vastus Medalis 1/4; Algo II = Algometer location #2: Vastus Lateralis 1/4; Algo III = Algometer location #3: Vastus Lateralis 1/2; G = group p-level; T = time p-level; G x T = interaction; q = quadratic p-level; L = linear p-level

### Isokinetic maximal voluntary contraction performance assessment

Table 4 reports the total work performed during the 3-repetition isokinetic flexion/extension MVC test. The overall MANOVA analysis revealed both a non-significant Wilks’ Lambda time interaction (*p* = 0.96) and a non-significant group x time effect (*p* = 0.75). The 3-repetition work summation univariate measures for extension (*p* < 0.001), flexion (*p* = 0.022), and total (*p* < 0.001) demonstrated significant changes over time, with the lowest work attained 60-min post-lift in all three measures. No aspect of the isokinetic strength recovery work performed demonstrated a significant group effect over time. While changes from pre-lift were not statistically significant between groups across time, the drop in 3-repetition summation of flexion (*p* = 0.21; *d* = 0.45), extension (*p* = 0.23; *d* = 0.45), and total work (*p* = 0.15; *d* = 0.55) performed 60-min post-lift may have been attenuated in TC compared to P based upon the moderate to large Cohen’s d effect size calculations. Average recovery decreases in flexion, extension, and total work performance from pre-lift values were 5, 19, and 14 % respectively in P, but only 2, 16, and 10 % in TC.

**Table 4:** Isokinetic Maximal Voluntary Contraction Knee Extension/Flexion Total Work Performance

Variable	Group	Baseline	Pre-Lift	60-min Post	24-hr Post	48-hr Post	Group Mean	p-value (GG)	p-value (WSC)
IK Total Ext Work (N)	P	1791±207	1682±235	1278±275	1408±296	1387±291	1509±90	*G=*0.870	
	TC	1757±378	1612±437	1298±422	1418±463	1352±383	1487±94	*T<*0.001*	*T*_*L*_ *<*0.001*
	Time Mean	1774±295	1648±340^†^	1288±345^†Ψ^	1413±376^†Ψ◊^	1370±331^†Ψ^		*G X T=*0.792	*G X T*_*q*_ *=*0.662
IK Total Flex Work (N)	P	971±126^	962±141^	913±174^	925±153^	910±113^^†^	936±58	*G=*0.181	
	TC	1073±252	1060±277	1064±225	1058±305	1010±289^†^	1053±61	*T=*0.087^§^	*T*_*L*_ *=*0.022*
	Time Mean	1020±199	1009±218	986±210	989±242	958±217^†Ψ^		*G X T=*0.663	*G X T*_*q*_ *=*0.206
IK Total Work (N)	P	2762±293	2644±354	2191±388	2333±407	2296±373	2445±141	*G=*0.646	
	TC	2829±611	2673±687	2363±603	2476±733	2362±644	2540±148	*T<*0.001*	*T*_*L*_ *<*0.001*
	Time Mean	2794±462	2658±527^†^	2273±498^†Ψ^	2401±577^†Ψ◊^	2328±509^†Ψ^		*G X T=*0.692	*G X T*_*q*_ *=*0.470

Individual group and time data expressed as means ± SD, while group effects are presented as means ± SEM. Data represents the isokinetic MVC knee extension and flexion performance each test session during the 10 day intervention. MANOVA analysis revealed overall Wilks' Lambda time (p<0.960) and group x time (p=0.748). Univariate ANOVA p-levels from MANOVA analysis are presented for each variable. Univariate ANOVA p-levels are listed first by the Greenhouse-Geisser (GG) analysis and then by the within-subjects contrasts (WSC) to demonstrate the potential shape of the time or group x time interaction with significance indicated by the following super/subscripts: * indicates p<0.05 p-level significance, § indicates p<0.10 p-level significance. LSD post hoc analysis is indicated by the following superscripts: ^ represents p<0.05 difference between groups, † represents p<0.05 difference from baseline value, Ψ represents p<0.05 difference from pre-lift, ◊ represents p<0.05 difference from 60-min post, # represents p<0.05 difference from 24-hr post. Ext = extension; Flex = flexion; IK = isokinetic; MVC = maximal voluntary contraction; G = group p-level; T = time p-level; G x T = interaction; q = quadratic p-level; L = linear p-level

### Markers of mechanical damage and physiological stress

**Fig. 4:**
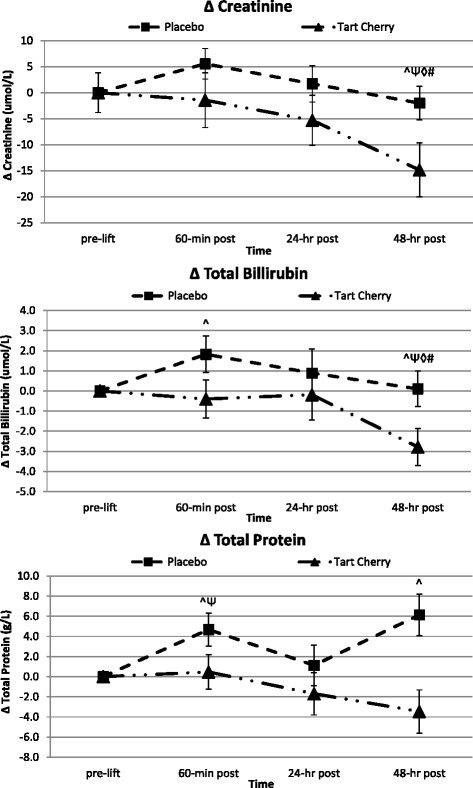
Markers of protein catabolism. Data expressed as means ± SE and significance indicated by the following super/subscripts: * indicates *p* < 0.05 p-level significance, § indicates *p* < 0.10 p-level significance. LSD post hoc analysis is indicated by the following superscripts: ^ represents *p* < 0.05 difference between groups, Ψ represents *p* < 0.05 difference from pre-lift, ◊ represents *p* < 0.05 difference from 60-min post, # represents *p* < 0.05 difference from 24-h post

**Fig. 5:**
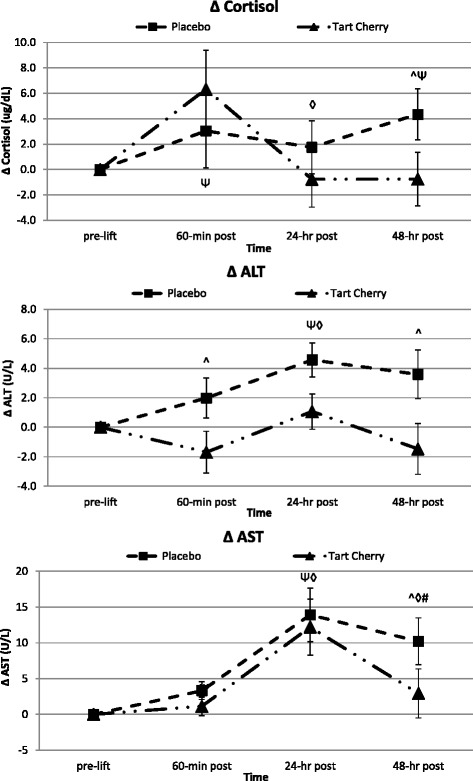
Markers of physiological stress and secondary indices of muscle damage. Data expressed as means ± SE and significance indicated by the following super/subscripts: * indicates *p* < 0.05 p-level significance, § indicates *p* < 0.10 p-level significance. LSD post hoc analysis is indicated by the following superscripts: ^ represents *p* < 0.05 difference between groups, Ψ represents *p* < 0.05 difference from pre-lift, ◊ represents *p* < 0.05 difference from 60-min post, # represents *p* < 0.05 difference from 24-h post

Table 5 presents the serum mechanical damage and physiological stress markers tested in the standard clinical safety panel. The overall MANOVA analysis revealed a significant overall Wilks’ Lambda time (*p* < 0.001) effect, but no difference between groups over time (*p* = 0.50). Univariate measures for uric acid (*p* < 0.001), total bilirubin (*p* = 0.026), creatinine (*p* = 0.003), and total protein (*p* < 0.001) demonstrated significant changes over time, peaking 60-min post-lift. CK (*p* = 0.025) and AST (*p* = 0.003) also significantly changed over time, peaking 24-h post-lift. Significant group differences over time and group by time changes from pre-lift were reported for creatinine (*p* = 0.030, delta *p* = 0.024) and total protein (*p* = 0.018, delta *p* = 0.006). Analyzing the change from pre-lift levels, significant differences across groups for creatinine (*p* = 0.007) and total protein (*p* = 0.004) were also evident. Serum creatinine increased on average 2 % over pre-lift values during the recovery in P, but actually decreased 7 % below pre-lift in TC. Serum total protein increased 2–9 % over pre-lift values during the recovery in P, but decreased 0–4 % below pre-lift in TC. Subsequent post-hoc analysis indicated a significantly attenuated TC creatinine response 48-h post-lift and a TC total protein level that never differed from pre-lift measures 60-min and 48-h post-lift compared to deviations in P (see Fig. 4). Increases in serum CK levels tended to be lower in TC over time compared to P (*p* = 0.10). Serum CK levels increased on average 135 % over pre-lift values during the recovery in P, but increased only 98 % in TC. No other significant differences between groups over time or deviations from normal human clinical ranges were observed for kidney/liver enzymes [e.g. AST (*p* = 0.16), ALT (*p* = 0.27)] or markers of protein catabolism [bilirubin (*p* = 0.12), BUN:creatinine ratio (*p* = 0.39), uric acid (*p* = 0.15)]. However, further post-hoc analyses revealed TC facilitated post-lift attenuations in bilirubin, AST, and ALT 48-h post-lift compared to P (see Figs. 4 and 5).

**Table 5:** Markers of Muscle Catabolism, Secondary Muscle Damage, and Physiological Stress

Variable	Group	Baseline	Pre-Lift	60-min Post	24-hr Post	48-hr Post	Group Mean	p-value (GG)	p-value (WSC)
AST (U/L)	P	31.85±9.65	27.47±6.40	30.79±7.88	41.39±13.31	37.67±10.33	33.83±2.19	*G=*0.919	
	TC	37.70±17.65	28.39±7.62	29.53±6.46	40.59±17.59	31.32±11.57	33.51±2.29	*T=*0.003*	*T*_*q*_ *=*0.759
	Time Mean	34.78±2.93	27.93±1.46^†^	30.16±1.51^Ψ^	40.99±3.23^Ψ◊^	34.50±2.28^Ψ#^		*G X T=*0.316	*G X T*_*L*_ *=*0.162
ALT (U/L)	P	33.81±18.74	32.21±12.26	34.19±13.60	36.78±14.97	35.80±13.84	34.56±4.19	*G=*0.455	
	TC	31.78±13.22	30.01±19.33	28.32±15.83	31.08±19.58	28.54±17.41	29.94±4.38	*T=*0.529	*T*_*L*_ *=*0.180
	Time Mean	32.79±3.41	31.11±3.34	31.26±3.07	33.93±3.61^Ψ◊^	32.17±3.27		*G X T=*0.521	*G X T*_*L*_ *=*0.266
Total Billirubin (umol/L)	P	8.00±3.67	7.25±3.47	9.08±5.31	8.14±3.82	7.35±3.21	7.96±1.01	*G=*0.395	
	TC	9.65±3.85	9.98±4.95	9.58±4.01	9.79±5.20	7.19±3.21	9.24±1.06	*T=*0.064^§^	*T*_*q*_ *=*0.026*
	Time Mean	8.83±0.78	8.61±0.89	9.33±0.99	8.97±0.95	7.27±0.67^†Ψ◊#^		*G X T=*0.306	*G X T*_*L*_ *=*0.125
Urea/BUN (mmol/L)	P	6.59±1.54	6.16±1.74	6.07±1.59	6.48±1.96	5.92±2.33	6.24±0.44	*G=*0.794	
	TC	6.49±1.39	6.75±1.30	6.40±1.33	6.59±1.83	5.83±1.66	6.41±0.46	*T=*0.061^§^	*T*_*L*_ *=*0.055^§^
	Time Mean	6.54±0.31	6.46±0.32	6.24±0.31	6.53±0.40	5.88±0.43^†Ψ#^		*G X T=*0.594	*G X T*_*q*_ *=*0.238
Creatinine (umol/L)	P	96.50±13.23	97.19±19.28	102.76±17.25^	98.91±21.16^	95.20±17.73^^◊^	98.11±4.05	*G=*0.273	
	TC	91.82±11.99	96.84±15.27	95.42±14.37	91.52±11.27	81.99±14.87^†Ψ◊#^	91.52±4.23	*T=*0.004*	*T*_*q*_ *=*0.003*
	Time Mean	94.16±2.64	97.02±3.65	99.09±3.33	95.21±3.58	88.60±3.43^†Ψ◊#^		*G X T=*0.178	*G X T*_*L*_ *=*0.030*
BUN/Creatinine Ratio	P	17.11±4.38	15.97±3.99	14.82±3.66	16.66±5.67	15.50±5.26	16.01±1.08	*G=*0.353	
	TC	17.65±3.76	17.51±3.83	16.82±3.74	17.85±4.35	17.64±3.80	17.50±1.13	*T=*0.214	*T*_*q*_ *=*0.225
	Time Mean	17.37±4.01	16.71±3.90	15.78±3.75^†Ψ^	17.23±5.00	16.53±4.64		*G X T=*0.721	*G X T*_*L*_ *=*0.388
Uric Acid (umol/L)	P	328±66	317±66	444±136	367±80	349±88	361.12±20.37	*G=*0.647	
	TC	306±52	310±54	470±117	350±93	302±79	347.44±21.28	*T<*0.001*	*T*_*q*_ *<*0.001*
	Time Mean	317±12	314±13	457±27^†Ψ^	358±18^†Ψ◊^	325±17^◊#^		*G X T=*0.298	*G X T*_*q*_ *=*0.146
CK (U/L)	P	468±288	322±243	451±267	967±700	855±981	613±82	*G=*0.487	
	TC	843±1156	259±146	355±198	770±470	415±213	529±86	*T=*0.025*	*T*_*q*_ *=*0.222
	Time Mean	656±172	291±42^†^	403±49^Ψ^	869±126^Ψ◊^	635±151^Ψ^		*G X T=*0.209	*G X T*_*L*_ *=*0.100^§^
Total Protein (mmol/L)	P	78.67±6.11	67.55±7.96^^†^	72.23±6.47^†Ψ^	68.67±9.45^†^	73.67±11.11^†Ψ#^	72.16±1.52	*G=*0.305	
	TC	80.53±4.60	74.13±2.62^†^	74.59±4.18^†^	72.43±6.72^†^	70.66±7.92^†Ψ◊^	74.47±1.59	*T<*0.001*	*T*_*q*_ *<*0.001*
	Time Mean	79.60±1.14	70.84±1.26^†^	73.41±1.15^†Ψ^	70.55±1.72^†^	72.17±2.03^†^		*G XT=*0.091^§^	*G X T*_*q*_ *=*0.018*

Individual group and time data expressed as means ± SD, while group effects are presented as means ± SEM. Data represents the response to muscle catabolism, mechanical damage, and physiological stress at each test session during the 10 day intervention. MANOVA analysis revealed overall Wilks' Lambda time (p<0.001) and group x time (p=0.504). Univariate ANOVA p-levels from MANOVA analysis are presented for each variable. Univariate ANOVA p-levels are listed first by the Greenhouse-Geisser (GG) analysis and then by the within-subjects contrasts (WSC) to demonstrate the potential shape of the time or group x time interaction with significance indicated by the following super/subscripts: * indicates p<0.05 p-level significance, § indicates p<0.10 p-level significance. LSD post hoc analysis is indicated by the following superscripts: ^ represents p<0.05 difference between groups, † represents p<0.05 difference from baseline value, Ψ represents p<0.05 difference from pre-lift, ◊ represents p<0.05 difference from 60-min post, # represents p<0.05 difference from 24-hr post. *AST* Aspartate aminotransferase, *ALT* Alanine aminotransferase, *BUN* blood urea nitrogen, *CK* Creatine kinase, *G* group p-level, *T* time p-level, *G x T* interaction, *q* quadratic p-level, *L* linear p-level

### Anabolic/catabolic hormone response markers

Table 6 shows the serum anabolic and catabolic hormone markers analyzed in response to exercise. The overall MANOVA analyses revealed both non-significant overall Wilks’ Lambda time (*p* = 0.29) and overall group changes across time (*p* = 0.46). Cortisol levels tended to change over time (*p* = 0.093). Serum cortisol levels were significantly different between groups over time (*p* = 0.029) and revealed how supplementation caused significant variability (*p* = 0.032) in cortisol changes from pre-lift levels across the recovery period. Serum cortisol levels increased on average 13 % over pre-lift values during the recovery in P, but only 8 % in TC. The delta post-hoc analysis revealed that TC cortisol levels were not different from pre-lift levels and were significantly lower at 48-h post-lift compared to rising cortisol levels in P (see Fig. 5).

**Table 6:** Anabolic/Catabolic Hormone Response

Variable	Group	Baseline	Pre-Lift	60-min Post	24-hr Post	48-hr Post	Group Mean	p-value (GG)	p-value (WSC)
Cortisol (ug/dL)	P	28.34±13.07^	23.33±8.80	26.37±7.33	25.08±10.21^	27.68±11.20^	26.16±2.18	*G=*0.167	
	TC	21.48±7.02	20.49±7.92	26.81±12.08^†^	19.72±5.56^◊^	19.74±6.82^◊^	21.65±2.28	*T=*0.093^§^	*T*_*L*_ *=*0.611
	Time Mean	24.91±2.22	21.91±1.75	26.59±2.06^Ψ^	22.40±1.74^◊^	23.71±1.96		*G X T=*0.170	*G X T*_*q*_ *=*0.029*
Testosterone (ng/mL)	P	9.86±3.46	9.37±3.45	9.25±2.92	10.17±3.55	9.94±3.42	9.72±0.75	*G=*0.025*	
	TC	7.24±2.22	7.17±2.00	6.97±1.95	7.20±2.00	6.91±1.70	7.10±0.79	*T=*0.428	*T*_*q*_ *=*0.427
	Time Mean	8.55±0.61	8.27±0.60	8.11±0.52	8.68±0.61	8.42±0.57		*G X T=*0.556	*G X T*_*L*_ *=*0.174
Test/Cort Ratio	P	0.037±0.009	0.043±0.014	0.037±0.013	0.045±0.019	0.040±0.018	0.040±0.003	*G=*0.538	
	TC	0.037±0.015	0.040±0.020	0.031±0.015	0.039±0.013	0.040±0.019	0.037±0.004	*T=*0.152	*T*_*L*_ *=*0.516
	Time Mean	0.037±0.012	0.042±0.017	0.034±0.014	0.042±0.016	0.040±0.018		*G X T=*0.815	*G X T*_*q*_ *=*0.196

Individual group and time data expressed as means ± SD, while group effects are presented as means ± SEM. Data represents the stress and sex hormone response at each test session during the 10 day intervention. MANOVA analysis revealed overall Wilks' Lambda time (p=0.293) and group x time (p=0.458). Univariate ANOVA p-levels from MANOVA analysis are presented for each variable. Univariate ANOVA p-levels are listed first by the Greenhouse-Geisser (GG) analysis and then by the within-subjects contrasts (WSC) to demonstrate the potential shape of the time or group x time interaction with significance indicated by the following super/subscripts: * indicates p<0.05 p-level significance, § indicates p<0.10 p-level significance. LSD post hoc analysis is indicated by the following superscripts: ^ represents p<0.05 difference between groups, † represents p<0.05 difference from baseline value, Ψ represents p<0.05 difference from pre-lift, ◊ represents p<0.05 difference from 60-min post, # represents p<0.05 difference from 24-hr post. Test/Cort = Testosterone/Cortisol ratio *G* group p-level, *T* time p-level, *G x T* interaction, *q* quadratic p-level; L = linear p-level

### Markers of free radical production and oxidative stress

Table 7 reports the serum markers of free radical production [reactive oxygen species (ROS) and reactive nitrogen species (RNS)] analyzed in response to exercise. The overall MANOVA analysis revealed no significant overall Wilks’ Lambda time (*p* = 0.32) interaction and no overall group differences over time (*p* = 0.76). None of the univariate measures for markers of free radical production, lipid peroxidation, and antioxidant capacity/activity (NT, TBARS, TAS, and SOD) reported main time effects or differences between groups over time.

**Table 7:** Markers of Free Radical Production and Oxidative Stress

Variable	Group	Baseline	Pre-Lift	60-min Post	24-hr Post	48-hr Post	Group Mean	p-value (GG)	p-value (WSC)
Nitrotyrosine (nM)	P	305±267	306±311	297±289	297±330	294±305	300±74	*G=*0.940	
	TC	303±193	319±254	277±190	275±202	283±203	292±78	*T=*0.280	*T*_*L*_ *=*0.146
	Time Mean	304±230	312±279	288±241	287±270	289±256^Ψ^		*G X T=*0.613	*G X T*_*L*_ *=*0.483
TBARS (uM)	P	7.84±2.69	7.27±2.91	8.60±2.67	8.03±3.34	8.43±3.74	8.03±0.81	*G=*0.913	
	TC	7.74±4.24	8.24±4.03	7.44±3.84	8.19±3.46	7.91±3.35	7.90±0.84	*T=*0.937	*T*_*L*_ *=*0.539
	Time Mean	7.79±3.44	7.73±3.44	8.05±3.26	8.10±3.32	8.18±3.49		*G X T=*0.569	*G X T*_*L*_ *=*0.649
TAS (mM)	P	2.90±1.34	2.83±1.77	3.02±1.85	2.95±1.47	2.80±1.81	2.90±0.39	*G=*0.752	
	TC	2.69±1.17	2.39±1.14	2.99±1.07	2.73±1.36	2.81±1.34	2.72±0.41	*T=*0.323	*T*_*q*_ *=*0.419
	Time Mean	2.80±1.23	2.62±1.48	3.01±1.49	2.84±1.39	2.81±1.57		*G X T=*0.706	*G X T*_*L*_ *=*0.442
SOD (U/mL)	P	0.58±0.07	0.59±0.05	0.59±0.09	0.56±0.08	0.58±0.06	0.58±0.02	*G=*0.668	
	TC	0.55±0.12	0.60±0.09	0.54±0.09	0.57±0.10	0.57±0.11	0.57±0.02	*T=*0.264	*T*_*q*_ *=*0.827
	Time Mean	0.56±0.10	0.60±0.07^†^	0.57±0.09	0.57±0.08	0.58±0.09		*G X T=*0.335	*G X T*_*L*_ *=*0.784

Individual group and time data expressed as means ± SD, while group effects are presented as means ± SEM. Data represents the response to reactive oxygen and nitrogen species production in addition to antioxidant activity at each test session during the 10 day intervention. MANOVA analysis revealed overall Wilks' Lambda time (p=0.321) and group x time (p=0.756). Univariate ANOVA p-levels from MANOVA analysis are presented for each variable. Univariate ANOVA p-levels are listed first by the Greenhouse-Geisser (GG) analysis and then by the within-subjects contrasts (WSC) to demonstrate the potential shape of the time or group x time interaction with significance indicated by the following super/subscripts: * indicates p<0.05 p-level significance, § indicates p<0.10 p-level significance. LSD post hoc analysis is indicated by the following superscripts: ^ represents p<0.05 difference between groups, † represents p<0.05 difference from baseline value, Ψ represents p<0.05 difference from pre-lift, ◊ represents p<0.05 difference from 60-min post, # represents p<0.05 difference from 24-hr post

*TBARS* Thiobarbituric acid reactive substances, *TAS* Total antioxidant status, *SOD* superoxide dismutase, *G* group p-level, *T* time p-level, *G x T* interaction, *q* quadratic p-level, *L* linear p-level

### Inflammatory response markers

Table 8 shows the serum inflammatory cytokine and chemokine markers analyzed in response to exercise. The overall MANOVA analysis revealed a significant overall Wilks’ Lambda time (*p* < 0.001) interaction, but a non-significant overall group x time effect (*p* = 0.30). Univariate measures for TNF-α (*p* = 0.001), IL-1β (*p* = 0.030), IL-6 (*p* = 0.023), and IL-8 (*p* = 0.018) demonstrated significant changes over time and from baseline measures, peaking 24-h post-lift. No significant differences between groups were observed over time for any of the inflammatory cytokines or chemokines.

**Table 8:** Pro-inflammatory Cytokines and Chemokines

Variable	Group	Baseline	Pre-Lift	60-min Post	24-hr Post	48-hr Post	Group Mean	p-value (GG)	p-value (WSC)
TNF-α (pg/mL)	P	2.82±1.33	3.30±1.59	3.33±1.88	2.98±1.45	2.85±1.41	3.05±0.36	*G=*0.212	
	TC	2.12±0.92	2.55±1.15	2.61±1.00	2.44±0.82	2.23±0.94	2.39±0.37	*T=*0.003*	*T*_*q*_ *=*0.001*
	Time Mean	2.47±0.24	2.92±0.29^†^	2.97±0.32^†^	2.71±0.25^◊^	2.54±0.25^Ψ◊^		*G X T=*0.873	*G X T*_*L*_ *=*0.649
IFN-γ (pg/mL)	P	14.92±31.51	18.91±44.08	18.99±44.51	21.33±51.78	21.56±51.82	19.14±9.41	*G=*0.365	
	TC	6.19±5.69	6.46±5.01	7.12±5.19	7.11±5.98	5.78±6.02	6.53±9.83	*T=*0.287	*T*_*q*_ *=*0.136
	Time Mean	10.56±4.83	12.68±6.70	13.06±6.77	14.22±7.87	13.67±7.88^#^		*G X T=*0.314	*G X T*_*L*_ *=*0.295
IL-1β (pg/mL)	P	0.77±0.32	0.84±0.35	0.87±0.39	0.83±0.38	0.83±0.35	0.83±0.10	*G=*0.048*	
	TC	0.75±0.35	0.75±0.34	0.82±0.26	0.85±0.30	0.82±0.34	0.80±0.10	*T=*0.047*	*T*_*q*_ *=*0.030*
	Time Mean	0.76±0.07	0.80±0.07^†^	0.85±0.07^†^	0.84±0.07	0.82±0.07		*G X T=*0.411	*G X T*_*q*_ *=*0.488
IL-2 (pg/mL)	P	1.86±1.86	2.00±2.18	1.97±2.16	1.91±2.15	1.87±2.09	1.92±0.71	*G=*0.956	
	TC	1.70±2.96	1.73±2.73	2.06±2.65	2.26±3.13	2.15±3.03	1.98±0.74	*T=*0.292	*T*_*L*_ *=*0.214
	Time Mean	1.78±0.51	1.87±0.51	2.02±0.50	2.08±0.56	2.01±0.54		*G X T=*0.230	*G X T*_*L*_ *=*0.163
IL-6 (pg/mL)	P	1.19±0.95	1.34±1.36	1.61±1.60	1.33±1.38	1.26±1.21	1.35±0.37	*G=*0.724	
	TC	0.87±1.34	1.09±1.31	1.54±1.38	1.20±1.49	1.07±1.43	1.16±0.39	*T=*0.020*	*T*_*q*_ *=*0.023*
	Time Mean	1.03±0.24	1.22±0.28	1.57±0.31^†Ψ^	1.27±0.30^†^	1.17±0.28^◊#^		*G X T=*0.761	*G X T*_*L*_ *=*0.512
IL-8 (pg/mL)	P	4.25±3.99	5.07±5.74	5.04±6.16	5.16±6.41	4.94±6.32	4.89±1.23	*G=*0.496	
	TC	2.95±1.56	3.45±1.75	4.14±1.29	4.02±1.78	3.74±1.91	3.66±1.28	*T=*0.042*	*T*_*q*_ *=*0.018*
	Time Mean	3.60±0.64	4.26±0.90^†^	4.59±0.95^†^	4.59±1.00	4.34±0.99		*G X T=*0.697	*G X T*_*L*_ *=*0.736
IL-12p70 (pg/mL)	P	2.27±4.13	3.03±6.51	3.05±6.63	3.01±6.42	2.78±5.68	2.83±1.33	*G=*0.577	
	TC	1.70±2.67	1.76±2.59	1.68±2.15	1.91±2.76	1.66±2.61	1.74±1.39	*T=*0.279	*T*_*q*_ *=*0.264
	Time Mean	1.99±0.73	2.39±1.05	2.37±1.05	2.46±1.05	2.22±0.94		*G X T=*0.377	*G X T*_*L*_ *=*0.351

Individual group and time data expressed as means ± SD, while group effects are presented as means ± SEM. Data represents the pro-inflammatory response at each test session during the 10 day intervention. MANOVA analysis revealed overall Wilks' Lambda time (p<0.001) and group x time (p=0.302). Univariate ANOVA p-levels from MANOVA analysis are presented for each variable. Univariate ANOVA p-levels are listed first by the Greenhouse-Geisser (GG) analysis and then by the within-subjects contrasts (WSC) to demonstrate the potential shape of the time or group x time interaction with significance indicated by the following super/subscripts: * indicates p<0.05 p-level significance, § indicates p<0.10 p-level significance. LSD post hoc analysis is indicated by the following superscripts: ^ represents p<0.05 difference between groups, † represents p<0.05 difference from baseline value, Ψ represents p<0.05 difference from pre-lift, ◊ represents p<0.05 difference from 60-min post, # represents p<0.05 difference from 24-hr post

*TNF-α* tumor necrosis factor alpha, *IFN-γ* interferon gamma, *IL* interleukin, *G* group p-level, *T* time p-level, *G x T* interaction, *q* quadratic p-level, *L* linear p-level

### Anti-inflammatory response markers

Table 9 presents the serum anti-inflammatory cytokine markers analyzed in response to exercise. The overall MANOVA analysis revealed a significant overall Wilks’ Lambda time (*p* < 0.001) interaction, but a non-significant overall effect of treatment over time (*p* = 0.45). Univariate measures for IL-4 (*p* = 0.001) and IL-7 (*p* = 0.033) reported significant main time effects with IL-13 levels approaching significance across time (*p* = 0.055), peaking 60-min post-lift. No significant group effects over time were reported for any of the anti-inflammatory cytokine markers.

**Table 9:** Anti-inflammatory Cytokines

Variable	Group	Baseline	Pre-Lift	60-min Post	24-hr Post	48-hr Post	Group Mean	p-value (GG)	p-value (WSC)
IL-4 (pg/mL)	P	6.03±3.18	7.86±5.06	8.26±5.22	7.44±4.95	7.83±5.10	7.48±1.05	*G=*0.273	
	TC	4.16±1.91	5.45±2.04	6.38±2.49	6.36±2.45	6.54±3.01	5.78±1.09	*T=*0.001*	*T*_*q*_ *=*0.001*
	Time Mean	5.10±0.55	6.65±0.82^†^	7.32±0.87^†^	6.90±0.83^†◊^	7.19±0.88^†^		*G X T=*0.421	*G X T*_*L*_ *=*0.405
IL-5 (pg/mL)	P	0.69±0.37	0.70±0.41	0.70±0.46	0.64±0.37	0.65±0.40	0.68±0.11	*G=*0.749	
	TC	0.75±0.43	0.72±0.40	0.71±0.37	0.77±0.43	0.70±0.42	0.73±0.12	*T=*0.716	*T*_*L*_ *=*0.325
	Time Mean	0.72±0.08	0.71±0.09	0.70±0.09	0.71±0.08	0.68±0.09		*G X T=*0.438	*G X T*_*L*_ *=*0.582
IL-7 (pg/mL)	P	4.73±2.19	5.41±2.49	5.94±3.71	5.52±2.75	5.46±2.57	5.41±0.63	*G=*0.414	
	TC	4.15±1.86	4.45±2.00	5.31±2.18	4.91±1.61	4.47±1.54	4.66±0.65	*T=*0.041*	*T*_*q*_ *=*0.033*
	Time Mean	4.44±0.43	4.93±0.48^†^	5.62±0.64^†^	5.21±0.48^†^	4.97±0.45^†^		*G X T=*0.843	*G X T*_*L*_ *=*0.757
IL-10 (pg/mL)	P	4.52±3.85	3.69±1.82	4.21±1.92	3.48±1.70	3.45±1.74	3.87±0.56	*G=*0.869	
	TC	2.77±1.74	3.49±2.03	6.02±5.70	3.33±2.54	3.07±2.54	3.74±0.59	*T=*0.103^§^	*T*_*q*_ *=*0.157
	Time Mean	3.65±0.63	3.59±0.40	5.12±0.87^Ψ^	3.41±0.45	3.26±0.45		*G X T=*0.182	*G X T*_*q*_ *=*0.120
IL-13 (pg/mL)	P	3.30±2.89	3.33±3.30	3.40±3.48	3.24±3.64	3.27±3.56	3.31±0.78	*G=*0.449	
	TC	1.96±1.74	2.26±1.66	2.75±2.09	2.78±2.18	2.45±2.34	2.44±0.81	*T=*0.393	*T*_*q*_ *=*0.055^§^
	Time Mean	2.63±0.50	2.80±0.55	3.08±0.61	3.01±0.63	2.86±0.64		*G X T=*0.402	*G X T*_*q*_ *=*0.133

Individual group and time data expressed as means ± SD, while group effects are presented as means ± SEM. Data represents the anti-inflammatory response at each test session during the 10 day intervention. MANOVA analysis revealed overall Wilks' Lambda time (p<0.001) and group x time (p=0.447). Univariate ANOVA p-levels from MANOVA analysis are presented for each variable. Univariate ANOVA p-levels are listed first by the Greenhouse-Geisser (GG) analysis and then by the within-subjects contrasts (WSC) to demonstrate the potential shape of the time or group x time interaction with significance indicated by the following super/subscripts: * indicates p<0.05 p-level significance, § indicates p<0.10 p-level significance. LSD post hoc analysis is indicated by the following superscripts: ^ represents p<0.05 difference between groups, † represents p<0.05 difference from baseline value, Ψ represents p<0.05 difference from pre-lift, ◊ represents p<0.05 difference from 60-min post, # represents p<0.05 difference from 24-hr post

*IL* interleukin, *G* group p-level, *T* time p-level, *G x T* interaction, *q* quadratic p-level, *L* linear p-level

### Clinical markers of immune-related complete blood counts

Table 10 displays immune response-related complete blood count markers analyzed in response to exercise. The overall MANOVA analysis demonstrated significant overall Wilks’ Lambda time interaction (*p* < 0.001), but a non-significant overall difference between groups over time (*p* = 0.68). Univariate measures for lymphocytes (*p* < 0.001) and MID (*p* < 0.001) demonstrated significant changes over time and from baseline measures, most depressed 60-min post-lift. WBC (*p* = 0.004) and GRAN (*p* = 0.003) also significantly changed over time, with lowest values 48-h post-lift. MID levels tended to be different between groups over time (*p* = 0.062). Significant group changes over time (*p* = 0.015) and differences in group effects from pre-lift levels over the recovery period (*p* = 0.013) were reported for lymphocytes. Lymphocyte levels decreased on average 23 % below pre-lift values during the recovery in P, but only 11 % in TC. Subsequent post-hoc analysis showed a significantly greater lymphocyte counts 24-h and 48-h post-lift in TC compared to P, but unlike those supplementing with P, TC lymphocyte counts returned to pre-lift values by the end of the recovery (48-h post-lift). A significant group effect over time was also reported for WBC (*p* = 0.020). WBC decreased on average 8 % below pre-lift values during the recovery in P, but only 3 % in TC. Post-hoc analysis demonstrated significant WBC differences pre-lift, 24-h, and 48-h post-lift between TC and P that became insignificant once changes were calculated from pre-lift levels.

**Table 10:** Markers of Immune-Related Complete Blood Counts

Variable	Group	Baseline	Pre-Lift	60-min Post	24-hr Post	48-hr Post	Group Mean	p-value (GG)	p-value (WSC)	
LYMPH (K/uL)	P	1.93±0.54	2.21±0.69	1.45±0.43^†Ψ^	1.86±0.71^^Ψ◊^	1.78±0.48^^Ψ◊^	1.85±0.20	*G=*0.311		
	TC	2.09±0.86	2.36±1.05^†^	1.49±0.58^†Ψ^	2.62±1.51^†Ψ◊^	2.15±0.78^◊#^	2.14±0.21	*T<*0.001*	*T*_*q*_ *=*0.136	
	Time Mean	2.01±0.15	2.28±0.18^†^	1.47±0.11^†Ψ^	2.24±0.24^◊^	1.97±0.13^Ψ◊^		*G X T=*0.143	*G X T*_*L*_ *=*0.015*	
WBC (K/uL)	P	5.88±1.23	6.61±2.21^	6.38±1.45	6.16±1.62^	5.75±1.61^	6.16±0.40	*G=*0.337		
	TC	5.54±1.25	7.18±2.03^†^	6.98±2.04^†^	7.40±2.17^†^	6.55±1.23^†#^	6.73±0.42	*T=*0.004*	*T*_*q*_ *=*0.004*	
	Time Mean	5.71±0.26	6.90±0.44^†^	6.68±0.37^†^	6.78±0.40^†^	6.15±0.30^Ψ^		*G X T=*0.207	*G X T*_*L*_ *=*0.020*	
MID (K/uL)	P	0.43±0.12	0.64±0.19	0.43±0.14	0.53±0.13	0.48±0.13	0.50±0.03	*G=*0.866		
	TC	0.42±0.10	0.61±0.24	0.38±0.10	0.60±0.19	0.55±0.16	0.51±0.03	*T<*0.001*	*T*_*L*_ *=*0.100^§^	
	Time Mean	0.43±0.02	0.63±0.05^†^	0.41±0.03^Ψ^	0.56±0.03^†◊^	0.51±0.03^†Ψ◊^		*G X T=*0.324	*G X T*_*L*_ *=*0.062^§^	
GRAN (K/uL)	P	3.50±0.97	3.76±1.47	4.51±1.20	3.78±1.33	3.48±1.32	3.80±0.33	*G=*0.577		
	TC	3.04±0.48	4.22±1.77	5.11±2.35	4.17±1.61	3.84±0.98	4.08±0.35	*T<*0.001*	*T*_*q*_ *=*0.002*	
	Time Mean	3.27±0.16	3.99±0.34^†^	4.81±0.38^†Ψ^	3.97±0.31^†◊^	3.66±0.03^◊^		*G X T=*0.332	*G X T*_*L*_ *=*0.119	
GM-CSF (pg/mL)	P	8.12±8.23	8.18±8.33	7.87±7.38	7.54±7.67	7.39±7.49	7.82±2.71	*G=*0.512		
	TC	10.37±11.40	10.51±10.83	10.52±10.48	10.67±11.19	10.09±10.89	10.43±2.83	*T=*0.446	*T*_*L*_ *=*0.277	
	Time Mean	9.24±2.06	9.34±2.00	9.20±1.88	9.10±1.99	8.74±1.93		*G X T=*0.624	*G X T*_*L*_ *=*0.450	

Individual group and time data expressed as means ± SD, while group effects are presented as means ± SEM. Data represents immune cellular response markers at each test session during the 10 day intervention. MANOVA analysis revealed overall Wilks' Lambda time (p<0.001) and group x time (p=0.684). Univariate ANOVA p-levels from MANOVA analysis are presented for each variable. Univariate ANOVA p-levels are listed first by the Greenhouse-Geisser (GG) analysis and then by the within-subjects contrasts (WSC) to demonstrate the potential shape of the time or group x time interaction with significance indicated by the following super/subscripts: * indicates p<0.05 p-level significance, § indicates p<0.10 p-level significance. LSD post hoc analysis is indicated by the following superscripts: ^ represents p<0.05 difference between groups, † represents p<0.05 difference from baseline value, Ψ represents p<0.05 difference from pre-lift, ◊ represents p<0.05 difference from 60-min post, # represents p<0.05 difference from 24-hr post

*LYMPH* lymphocytes, *WBC* white blood cell, *MID* mid-range absolute count, *GRAN* granulocyte absolute count, *GM-CSF* granulocyte-macrophage colony-stimulating factor, *G* group p-level, *T* time p-level, *G x T* interaction, *q* quadratic p-level, *L* linear p-level

## Discussion

This is the first study to investigate the effect of freeze dried Montmorency tart cherry skin powder on acute resistance exercise performance and recovery. It was hypothesized that supplementation with this novel powdered tart cherry skin supplement surrounding an acute bout of intense resistance exercise would reduce perceptions of muscle soreness, markers of muscle damage, oxidative stress, and inflammation, thus better maintaining subsequent performance within the first 48-h of recovery. The results of the present study demonstrate that this powered tart cherry formulation is effective in promoting decreased perceptions of muscle soreness following intense resistance exercise. Tart cherry powder reduced perceptions of muscle soreness in the distal vastus medalis and lateralis. In accordance with decreased perceptions of muscle soreness, tart cherry powder supplementation reduced serum markers of muscle catabolism and physiological stress over the 48-h post-lift period compared to placebo. As a result of the resistance exercise, markers of oxidative stress, lipid peroxidation, and antioxidant activity did not significantly change over time as was not affected by differences in supplementation. The inflammatory response deviated from pre-lift values during the recovery period, but the change was not different between supplement groups.

The attenuation of quadriceps muscle soreness in conjunction with reduced hemodynamic markers of muscle catabolism following the resistance training bout indicates that the consumption of tart cherry powder may have dampened the effects of the secondary muscle damage response. The initial injury to the muscle microstructure is defined by a mechanical disruption of the myofibrils, particularly in response to high volume and intensity eccentric exercise [9, 44, 56]. The damage to the muscle microstructure triggers a local inflammatory response characterized by infiltration of fluid, plasma proteins, and free radicals that all exacerbate the initial mechanical muscle damage, creating a secondary injury incidence [9, 44, 57–60]. Decreased perceptions of quadriceps muscle soreness and muscle catabolism indices reveal that supplementation with powdered tart cherry may aid in blunting the secondary muscle damage effects compared to placebo.

The apparent beneficial effect of tart cherry powder supplementation on the perception of muscle soreness after a resistance exercise bout is consistent with some of the previously published findings. Connolly et al. [20] reported that consumption of a Montmorency tart cherry juice in healthy college-aged males significantly reduced pain in the elbow flexors after a bout of eccentric exercise using a visual analog scale (VAS). Peak muscle pain was achieved 24-h post-exercise in the tart cherry group compared to a continued increase in pain 48-h post-exercise in the placebo group. In contrast, Connolly et al. [20] reported no difference in pressure pain threshold (PPT) scores between the two experimental groups. Using a strenuous single leg knee extension protocol in a cohort of well-trained male athletes, Bowtell et al. [4] demonstrated a trend in post-exercise muscle pain reduction via PPT with Montmorency tart cherry juice supplementation up to 48-h post-exercise compared to placebo. The positive results in the current study are similar to previous tart cherry supplementation research findings, showing that powdered tart cherry supplementation significantly attenuates muscle soreness perceptions throughout the 48-h post-lift recovery compared to placebo. Slight differences in study outcomes may lie in the disparity of muscle pain or soreness measures, tart cherry supplements, quantity of musculature involvement, and/or exercise modality. Measurement of muscle soreness perception in the present study utilizing both an algometer and a GRPS was implemented to help ameliorate the purely subjective nature of a VAS as the only measure of pain or soreness.

The attenuation in post-lift muscle soreness with powdered tart cherry supplementation may be described by underlying catabolic mechanisms. Similar to the results of the current study, Bowtell et al. [4], following exhaustive leg extension exercise, demonstrated a faster post-exercise recovery of knee extensor force coupled with a trend for a smaller CK percent change from pre-exercise levels up to 48-h post-exercise with tart cherry supplementation compared to placebo [43]. Following a treadmill incremental exercise protocol with thoroughbred horses, Ducharme et al. [61] reported an attenuation trending toward significance in CK levels during exercise recovery when supplementing with a tart cherry juice blend compared to placebo. Similar to the post-lift attenuations of ALT (60-min and 48-h post) and AST (48-h post) as secondary markers of muscle damage in the current study, Ducharme et al. [61] reported that AST was significantly mitigated during and following an incremental exercise protocol in the tart cherry-treated horses compared to placebo [43]. In an endurance-based crossover study examining the effects of acute ibuprofen (8400 mg) ingestion surrounding a 45-min downhill treadmill run, Donnelly et al. [62] reported an increase in serum CK, AST, and lactate dehydrogenase post-run with maximal activity of CK and AST occurring at 24-h post-run, while lactate dehydrogenase, creatinine, and urea peaked 6-h post-run. Donnelly et al. [62] revealed that serum CK and urea levels were significantly higher after ibuprofen supplementation compared to placebo throughout the post-run recovery. Analyzing hemodynamic clinical chemistry makers before, 4-h, and 24-h post-Boston marathon, Kratz et al. [63] reported significant increases in total CK, AST, ALT, total protein, uric acid, and creatinine 4-h post-race and confirmed that CK, creatinine, uric acid, ALT, and AST remained significantly elevated over pre-race values 24-h post-race. The attenuation of these markers in the current study, demonstrates beneficial powdered tart cherry supplementation effects on the post-resistance exercise catabolic, muscle damage, and stress response.

Acute, intensive resistance exercise (>60 % 1-RM) has been reported to significantly increase muscular mechanical trauma as the initial phase of injury [4, 64] coupled with a secondary inflammatory injury phase that occurs as a result of plasma protein and inflammatory cell infiltration of the damaged tissue [4, 9, 57]. Howatson et al. [44] reported significantly lower IL-6 concentrations after running a marathon that coincided with quicker recovery of knee extensor maximal strength following the marathon in Montmorency tart cherry juice supplemented subjects compared to placebo [44]. As an inflammatory marker, IL-6 was significantly attenuated immediately post-race with tart cherry consumption compared to placebo [43, 44]. In a strength-based supplement study with resistance trained subjects, Bowtell et al. [4] was also not able to detect any significant effects on IL-6 as a marker of inflammation. Further, Trombold et al. [65] reported similar non-significant inflammatory (IL-6) findings in a study involving non-resistance trained subjects performing repeated bouts of eccentric elbow flexor muscle contractions with consumption of pomegranate-derived ellagitannin [4]. Supplementation in the current study did not affect the inflammatory response to muscular trauma as markers peaked 60-min post-lift with no response differences between supplementation groups. Attenuation of muscle soreness perceptions and markers of muscle damage are not likely attributed to changes in inflammation following a single resistance exercise bout. Differences in results between studies are likely attributed to variation in intensity, duration, and modality of the aforementioned exercise protocols.

The increase in inflammatory-related cytokines and chemokines such as TNF-α, IL-1β, IL-6, and IL-8 during and immediately following exercise has been shown in previous research [66–68] and closely resembles the inflammatory response in the current study. As described in previous literature, the increase in serum IL-6 within both groups 60-min post-lift in the current study can likely be attributed to the large release of muscle-derived IL-6 as a result of the contracting muscle fibers [66, 69]. The influx of muscle-derived IL-6 within the systemic circulation may be one of the triggers for subsequent cortisol release in response to exercise-induced stress [66, 70]. Glucocorticoids, specifically cortisol, and anti-inflammatory cytokines such as IL-4, IL-10, and IL-13 are released during strenuous exercise and typically demonstrate an immunosuppressive influence to help modulate the immune response balance [71]. However, extended immunodepression due to elevated plasma cortisol during the post-exercise period, particularly following long bouts of intense training in the fasted state [66], may hinder recovery and subsequent performance. Previous research conducted by McAnulty et al. [72] in trained endurance athletes reported a significant cortisol increase over time, but no significant differences in immediate and 1.5-h post-race cortisol levels with 2-month vitamin E supplementation compared to placebo. Comparing 14-d supplementation of *N*-acetyl-cysteine (NAC), epigallocatechin gallate (EGCG), or a placebo in active males surrounding a single bout of eccentric knee-extensor exercise, Kerksick et al. [73] found a significant decrease in serum cortisol levels 6-h post-exercise, but no differences between groups up to 72-h post-exercise despite a significant blunting of muscle soreness perception 24-h post exercise in the two supplement groups compared to placebo. The current study demonstrated an increase in cortisol levels 60-min post-lift compared to pre-lift in both groups. However, unlike the cortisol and muscle soreness perception results reported by Kerksick et al. [73], the current study demonstrated significantly elevated cortisol levels and muscle soreness in the placebo group compared to the powdered tart cherry group 24-h and 48-h post-lift.

The placebo group elevations in serum cortisol levels in the latter recovery period may be linked to differences in the immune response and the degree of secondary muscle injury. Previous research by McCarthy et al. [74] and Robson et al. [75] demonstrate that in response to repetitive muscular contraction during and immediately after acute, intense exercise, there is a large increase in circulating neutrophils (GRAN) and lymphocytes. According to data collected by Robson et al. [75], plasma neutrophil levels tend to peak 2–3 hours post-exercise, but remain elevated above pre-exercise levels for up to 10-h post-exercise due to elevations in plasma cortisol (e.g. perception of stress) that facilitate the release of neutrophils from the bone marrow. The current study demonstrated a plasma neutrophil (GRAN) peak 60-min post-lift, but effects of supplementation could not be determined as levels returned to pre-lift concentrations by 24-h. Unlike the neutrophil response to acute exercise, lymphocytes respond in a biphasic pattern, where plasma levels are significantly elevated during and immediately post-exercise followed by a drop below pre-exercise levels during the initial recovery period due to a efflux from the blood circulation, and a slow return to baseline levels as equilibrium is reached again [71, 75]. Robson et al. [75] demonstrated that serum cortisol levels influence both the influx of neutrophils and efflux of lymphocytes from systemic circulation. The results of the current study demonstrated that lymphocyte levels in the placebo group never returned to pre-lift levels during the 48-h recovery. This may be linked to the elevated plasma cortisol levels in the placebo group representing an increased physiological perception of exercise stress 48-h post-lift compared to pre-lift and powdered tart cherry group levels. As previously mentioned, the secondary injury phase occurs as a result of significant plasma protein and inflammatory cell influx within the damaged tissue [4, 9, 57]. Powdered tart cherry supplementation may have aided in reducing secondary muscular injury through an initial dampening of the immune response paired with the attenuated cortisol response later in exercise recovery. However, immune cell count changes in response to supplementation and the resistance exercise stimulus are speculative due to the lack of cellular count data corresponding to typical patterns of immune cell responses during and immediately following the barbell back squat challenge.

The increase in antioxidant bioavailability from tart cherries containing high levels of flavonoids and anthocyanins [76, 77] has been hypothesized to be one of the main benefits of tart cherry supplementation. Antioxidant bioavailability is important in maintaining adequate redox balance to support the endogenous antioxidant systems following excessive ROS-producing strenuous exercise. Ducharme et al. [61] investigated the effects of Montmorency tart cherry juice supplementation on oxidative stress in thoroughbred horses following a stepwise incremental treadmill running protocol [43]. Overall indications of oxidative stress as a result of exercise were reported through significant elevations in lipid hydroperoxidation (TBARS) [61], but similar to the current study, no differences between groups were shown. In an endurance study examining the effects of tart cherry juice supplementation on oxidative stress following a marathon run, Howatson et al. [44] demonstrated significantly lower TBARS levels in the tart cherry supplemented group versus placebo 48-h post-marathon. As a highly reactive oxide metabolite of nitric oxide, peroxynitrate-bound tyrosine residues forming nitrotyrosine (NT) [78] were measured by Sureda et al. [79] following supplementation of vitamin C + E surrounding a half-marathon. Suerda et al. [79] reported a significant increase in both NT immediately post-race and 3-h post-race in the placebo group compared to the vitamin C + E supplemented group, indicating that antioxidant supplementation may have an attenuating effect on the oxidation of nitrogen-containing compounds surrounding endurance exercise. Contrarily, both Bowtell et al. [4] and the current study found no difference between supplementation groups with regard to oxidation of nitric oxide (NT) in the post-strength exercise period. A difference in outcome may be attributable to the difference in exercise modality and thus the minimal reliance on aerobic metabolic demands during resistance exercise. Further, evidence in the literature utilizing lipid peroxidation (TBARS) analyses have presented a potential lack of oxidative damage detection specificity in human studies that may also explain the variability in results between the current and previous studies [11, 43, 80, 81].

In a study analyzing the recovery from a marathon run, Howatson et al. [44] analyzed plasma TAS, as a measure of antioxidant or antiradical activity [82], and found that TAS fell below baseline 48-h post-marathon in the placebo group [43]. Unlike the tart cherry group, the placebo group failed to maintain TAS or redox balance following the endurance exercise, demonstrating possible antioxidant effectiveness on excessive ROS production with bouts of endurance exercise [43]. Childs et al. [83] analyzing TAS following arm eccentric exercise-induced injury demonstrated significantly greater TAS levels with vitamin C + NAC supplementation compared to placebo 48-h through 7-d post-exercise in healthy, untrained males. A resistance training-related study conducted by Lafay et al. [84] investigating the effects of a polyphenol-rich grape extract on repeated jumping force capacity, found a significant jumping force capacity improvement in the grape extract group over placebo. However, similar to the current study, Lafay et al. [84] found no significant difference in SOD activity between the groups. The current resistance training study also did not demonstrate any significant change in TAS activity across time or between groups possibly attributed to differences in study population and the low metabolic cost associated with resistance/eccentric exercise versus endurance exercise.

The strengths of this particular study revolve around the analysis of a large cohort of muscle soreness, performance, muscle damage, inflammatory, and oxidative damage measures to contribute a comprehensive analysis to the existing body of published literature. This type of comprehensive hemodynamic analysis has not been conducted in a tart cherry supplement study paired with resistance exercise. Further, the current study employed one of the largest subject cohorts among recent studies examining polyphenolic, vitamin, or NSAID supplementation effects on performance and related hemodynamic markers. The utilization of this supplement within the free-living resistance trained population demonstrated its effectiveness under normative training, diet, and performance conditions. The ease associated with consuming an encapsulated powdered supplement versus a potentially inconvenient or unpalatable juice most likely enhanced subject compliance with supplementation. Potential limitations and weakness of the current study should also be considered. The placebo-control matched design of this study was effective in equalizing study subject exposure to the resistance protocol irrespective of supplement group, however compared to a cross-over design, there might have been some variability associated with subject pairing. Differences in resistance training experience and overall state of training beyond the study inclusion/exclusion criteria may also have been a source of variability in study cohort recruitment. Due to the large number of hemodynamic markers measured in this study, the five selected time points of blood draws over the course of the experimental period may have not captured the entire pharmacokinetic profile for each marker. Despite controlling 48-h training activity and NSAID intake with a 10-h fast before each session, nutritional intake and hydration status of each subject was not controlled nor tested. Differences in nutrition and hydration among study subjects could have been a potential source of hemodynamic marker or performance measure variability in a cohort of this size. Further, the 48-hour NSAID ingestion limitation may not have been a sufficient washout time for all NSAID-type medications considering the large range in reported half-life of common NSAIDs (1.6 to 30-h). The major overriding strengths of this study are that this is the first practical study to be conducted surrounding an acute bout of strength exercise incorporating significant muscle mass recruitment and it is the first study to be conducted utilizing a powdered form of cherries rather than a juice or concentrate.

## Conclusions

The current study demonstrated that consumption of a Montmorency powdered tart cherry supplement 7-d before, the day of, and 2-d after completing a single bout of high volume, high-intensity resistance exercise, appears to be an effective dietary supplement in reducing muscle soreness across the most biomechanically loaded region of the quadriceps near the distal patellar attachment and markers of muscle catabolism in resistance trained individuals. Short-term supplementation with powdered tart cherry surrounding a single bout of resistance training did not demonstrate any definitive effect on markers of oxidative damage or inflammation. Due to the inconclusive oxidative damage and inflammatory evidence, mechanisms of short-term powdered tart cherry and other related phytochemical-containing nutritional supplements surrounding bouts of high intensity, anaerobic and resistance exercise need to be further investigated. Additional examination of powdered tart cherry supplementation with other forms of exercise that are known to promote a more pronounced effect on inflammation and oxidative stress (e.g. endurance exercise) is also needed. However, the initial effectiveness in reducing perceptions of muscle soreness and markers of muscle catabolism in resistance-trained men demonstrates that powdered tart cherry supplementation provides similar benefits as previously studied tart cherry juices or concentrates following acute bouts of lower body strength-based exercise.
